# Psychological impact of 2019 novel coronavirus (2019-nCoV) outbreak in health workers in China

**DOI:** 10.1017/S0950268820001090

**Published:** 2020-05-20

**Authors:** Dandan Sun, Dongliang Yang, Yafen Li, Jie Zhou, Wenqing Wang, Quanliang Wang, Nan Lin, Ailin Cao, Haichen Wang, Qingyun Zhang

**Affiliations:** 1 Department of Cardiology of Affiliated Hospital of Jining Medical University, 89# Guhuai Road, Rencheng district, Jining City 272000, Shandong Province, China; 2 Cangzhou Medical College, Cangzhou, Hebei, China; 3 Party Committee Office of Affiliated Hospital of Jining Medical University, 89# Guhuai Road, Rencheng district, Jining City 272000, Shandong Province, China

**Keywords:** 2019 Novel coronavirus, COVID-19, health workers, psychological

## Abstract

The first case of 2019-nCoV pneumonia infection occurred in Wuhan, Hubei Province, South China Seafood Market in December 2019. As a group with a high probability of infection, health workers are faced with a certain degree of psychological challenges in the process of facing the epidemic. This study attempts to evaluate the impact of 2019-nCoV outbreak on the psychological state of Chinese health workers and to explore the influencing factors. During the period from 31 January 2020 to 4 February 2020, the ‘Questionnaire Star’ electronic questionnaire system was used to collect data. The 2019-nCoV impact questionnaire and The Impact of Event Scale (IES) were used to check the psychological status of health workers in China. A total of 442 valid data were collected in this study. Seventy-four (16.7%) male and 368 (83.3%) female individuals participated in this study. The average score of high arousal dimension was 5.15 (s.d. = 4.71), and the median score was 4.0 (IQR 2.0, 7.0). The average score of IES was 15.26 (s.d. = 11.23), and the median score was 13.5 (IQR 7.0, 21.0). Multiple regression analysis showed that there were critical statistical differences in high arousal scores among different gender groups (male 3.0 *vs.* female 5.0, *P* = 0.075). Whether being quarantined had significant statistical differences of IES scores (being quarantined 16.0 *vs.* not being quarantined 13.0, *P* = 0.021). The overall impact of the 2019-nCoV outbreak on health workers is at a mild level. Chinese health workers have good psychological coping ability in the face of public health emergencies.

## Introduction

The first case of 2019-nCoV pneumonia infection occurred in Wuhan, Hubei Province, South China Seafood Market in December 2019 [1]. Due to the large-scale population movement during the Spring Festival, the pneumonia began to spread in various provinces and regions of China [2–5]. As of 4 February 2020, China had 20 471 confirmed cases, 23 214 suspected cases, 2788 critically ill cases, 425 deaths and 637 cured cases. According to the data, the number of 2019-nCoV pneumonia patients has far exceeded the severe acute respiratory syndrome (SARS) in 2003 [6]. As front-line workers in this battle, some doctors and nurses have been infected while taking care of patients infected with 2019-nCoV pneumonia. However, a large number of health workers from various provinces in China have taken the initiative to sign up for support in Hubei Province. As a group with a high probability of infection, health workers are faced with a certain degree of psychological challenges in the process of facing the epidemic [7]. Freeman MP wrote that the health workers are having an extraordinarily difficult time and the healthcare systems become increasingly overloaded [8]. Also, during the MERS outbreak, healthcare workers who performed MERS-related tasks scored significantly higher on the total IES-R and its subscales [9]. However, currently, there are few research studies on the mental health status of health workers during this novel coronavirus outbreak, so this study attempts to evaluate the impact of 2019-nCoV outbreak on the psychological state of Chinese health workers and to explore the influencing factors.

## Methods

### Study population and data collection

This study is a cross-sectional study. During the period from 31 January 2020 to 4 February 2020, the ‘Questionnaire Star’ electronic questionnaire system was used to collect data. ‘Questionnaire Star’ is an application dedicated to send electronic questionnaires. Researchers can design different options for each question for respondents to choose, and respondents can use Wechat or web page to answer. The results of the survey can eventually form an Excel data table. The questionnaire was filled out anonymously. The inclusion criteria of the subjects were as follows: age ⩾18 years old; working in hospital, including medical treatment, nursing, medical technology, administration, logistics; working in mainland China. A total of 450 questionnaires were collected, of which 442 were valid. Eight invalid questionnaires were excluded because of incomplete data (three questionnaires), response time of less than 90 s (three questionnaires) and those who have inconsistent answers to the same questions set in this questionnaire (two questionnaires). This study has been approved by the Ethics Committee of the Affiliated Hospital of Jining Medical University (NO: 2020C011).

### Study instruments

#### General information questionnaire

The general data and indicators of the subjects collected in this study include hospital level (Grade 3A hospital, tertiary hospital, secondary hospital, primary hospital, etc.), type of medical institution (public hospital, non-public hospital), hospital type (general hospital, traditional Chinese medicine hospital, integrated traditional Chinese and western medicine hospital, specialist hospital, etc.), age (⩽25 years old, 26–35 years old, 36–45 years old, 46–55 years old, ⩾56 years old), gender, job title (doctors, nursing staff, administrative and logistics staff, etc.), working years (⩽3 years, 3–5 years, 6–8 years, ⩾9 years), marital status (unmarried, married, divorced), number of children, living conditions (living with family members, dormitory, living alone), whether contact with 2019-nCoV confirmed patients, whether contact with suspected 2019-nCoV patients, whether being quarantined, whether participated in SARS treatment in 2003.

#### 2019-nCoV impact questionnaire

This questionnaire is compiled by the researchers and consists of nine items, each of which should be answered with Yes or No. The nine items of the questionnaire include:(1) My work puts me at great risk. (2) I feel more pressure at work than before. (3) I can accept the risk of exposure to or care for patients with the 2019-nCoV. (4) I am afraid of being infected with the 2019-nCoV. (5) I can ensure that my protective measures are effective enough. (6) If I am infected with the 2019-nCoV, I am confident that I can recover. (7) The 2019-nCoV outbreak makes me feel the urge to resign. (8) Because of working in the hospital, my family or friends are worried that they might get infected through me. (9) People stay away from me because of the nature of my work.

#### Impact of event scale

The Impact of Event Scale (IES) is used to evaluate the stress disorder level of threatening or catastrophic psychological trauma caused by unexpected events. The scale was first developed by Horowitz in 1979 [10]. The IES has been translated into Chinese version and has a good internal consistency reliability in Chinese population [11] (Cronbach’ s *α* coefficient is 0.87−0.92). The scale includes 22 items in three dimensions: intrusion symptoms (eight items), avoidance symptoms (eight items) and high arousal symptoms (six items). Each item uses the Likert 5-level score (0 = never, 1 = rarely, 2 = sometimes, 3 = often, 4 = always). The scores of invasive symptoms and avoidance symptoms can predict the severity of response to traumatic events. The score range of IES scale was 0–8 as subclinical, 9–25 as mild, 26–43 as moderate and 44–88 as severe.

### Statistical analysis

The counting data in this study are described by frequency and percentage. The measurement data are first tested for normality, and it is found that the measurement data in this study do not conform to the normal distribution, so the median and quartile are used to describe them. *W*−*H* method was used to compare the IES score and high arousal score between groups. Then the IES score and high arousal score were used as dependent variables to analyse the multiple regression model. The statistical software uses R software (version 3.6.1).

## Results

### Characteristics of respondents

A total of 442 valid data were collected in this study. Seventy-four (16.7%) male and 368 (83.3%) female individuals participated in this study. A total of 53 doctors, 348 nurses, 18 administrative and logistics staff and 23 other types of health workers participated in this study. In all, 337 subjects lived with their families, 27 subjects lived in dormitories and 38 subjects lived alone (Table 1).

**Table 1.** Comparison of IES and high arousal scores among different groups of health workers

Item	*N*（%）	Score of IES, median (IQR)	*P* value of IES	Score of high arousal, median (IQR)	*P* value of high arousal
Hospital level			0.123		0.102
Grade 3A hospital	369 (83.5)	13.0 (7.0, 21.0)		4.0 (1.0, 7.0)	
Tertiary hospital	26 (5.9)	12.0 (6.5, 15.0)		5.0 (2.25,5.75)	
Secondary hospital	30 (6.8)	13.5 (6.5, 18.25)		5.0 (1.0, 7.0)	
Primary hospital	13 (2.9)	20.0 (15.0, 25.0)		6.0 (4.0, 8.0)	
Others	4 (0.9)	8.5 (7.25, 10.5)		13.5 (12.5, 15.25)	
Type of medical institution			0.364		0.451
Public hospital	427 (96.6)	13.0 (7.0, 21.0)		4.0 (2.0, 7.0)	
Non-public hospital	15 (3.4)	17.0 (10.0, 22.5)		4.0 (0.5, 6.0)	
Hospital type			0.301		0.366
General hospital	396 (89.6)	13.0 (7.0, 21.0)		4.0 (1.75, 7.0)	
Traditional Chinese medicine hospital	15 (3.4)	11.0(6.0,14.5)		3.0 (0.0, 6.0)	
Integrated traditional Chinese and western medicine hospital	5 (1.1)	19.0 (16.0,26.0)		10.0 (5.0, 13.0)	
Specialist hospital	24 (5.4)	15.0 (5.0, 23.0)		4.5 (2.0, 8.0)	
Others	2 (0.5)	5.0 (4.0, 6.0)		7.5 (6.25, 8.5)	
Age			0.018		0.016
⩽25	36 (8.1)	11.0 (7.75, 26.0)		3.0(2.0, 7.25)	
26–35	261 (59.0)	14.0 (7.0, 21.0)		4.0 (2.0, 7.0)	
36–45	114 (25.8)	12.0 (6.25, 17.0)		4.0 (1.0, 6.0)	
46–55	28 (6.3)	19.5 (14.5, 26.0)		7.0 (4.75, 9.5)	
⩾56	3 (0.7)	29.0 (16.5, 32.5)		5.0 (4.5, 7.5)	
Gender			0.873		0.077
Male	74 (16.7)	13.5 (8.0, 18.0)		3.0 (1.0, 5.75)	
Female	368 (83.3)	13.5 (7.0, 22.0)		5.0 (2.0, 7.0)	
Job title			0.159		0.156
Doctors	53 (12.0)	16.0 (11.0, 22.0)		5.0 (3.0, 8.0)	
Nursing staff	348 (78.7)	13.0 (7.0, 20.25)		4.0 (1.0, 7.0)	
Administrative and logistics staff	18 (4.1)	13.0 (6.25, 26.5)		5.0 (1.25, 9.0)	
Others	23 (5.2)	15.0 (5.5, 18.0)		6.0 (2.5, 7.5)	
Working years			0.162		0.379
＜3	75 (17.0)	11.0 (7.0, 19.0)		3.0 (2.0, 7.0)	
3–5	66 (14.9)	15.0 (9.0, 22.75)		5.0 (2.0, 8.0)	
6–8	61 (13.8)	16.0 (9.0, 23.0)		5.0 (2.0, 7.0)	
⩾9	240 (54.3)	13.0 (6.75, 19.0)		4.0 (1.0, 7.0)	
Marital status			0.738		0.560
Unmarried	94 (21.3)	11.5 (7.0, 18.75)		3.5 (1.0, 7.0)	
Married	339 (76.7)	14.0 (7.0, 21.0)		4.0 (2.0, 7.0)	
Divorced	9 (2.0)	16.0 (5.0, 34.0)		5.0 (1.0, 12.0)	
Number of children			0.330		0.136
0	137 (31.0)	12.0 (7.0, 23.0)		4.0 (2.0, 7.0)	
1	203 (45.9)	15.0 (8.0, 22.0)		5.0 (2.0, 7.0)	
2	98 (22.2)	12.0 (6.0, 19.0)		4.0 (1.0, 6.0)	
⩾3	4 (0.9)	16.0 (12.0, 19.0)		5.0 (3.5, 6.75)	
Living conditions			0.915		0.581
Living with family members	377 (85.3)	14.0 (7.0, 21.0)		4.0 (2.0, 7.0)	
Dormitory	27 (6.1)	11.0 (8.5, 17.5)		3.0 (1.0, 6.0)	
Living alone	38 (8.6)	11.0 (7.0, 22.0)		4.0 (2.0, 6.75)	
Whether contact with 2019-nCov confirmed patients			0.327		0.303
Yes	70 (15.8)	15.0 (8.25, 22.0)		5.0 (2.0, 7.0)	
No	372 (84.2)	13.0 (7.0, 20.0)		4.0 (1.0, 7.0)	
Whether contact with suspected 2019-nCov patients			0.273		0.279
Yes	161 (36.4)	14.0 (8.0, 22.0)		5.0 (2.0, 7.0)	
No	281 (63.6)	13.0 (7.0, 19.0)		4.0 (1.0, 7.0)	
Whether being quarantined			0.042		0.068
Yes	29 (6.6)	16.0 (11.0, 27.0)		6.0 (3.0, 9.0)	
No	413 (93.4)	13.0 (7.0, 20.0)		4.0 (1.0, 7.0)	
Whether participated in SARS treatment in 2003			0.133		0.153
Yes	28 (6.3)	16.0 (7.75, 25.25)		6.0 (2.0, 8.25)	
No	414 (93.7)	13.0 (7.0, 20.0)		4.0 (1.0, 7.0)	

### Results of 2019-nCoV impact questionnaire

After the outbreak of 2019-nCoV, 395 (89.4%) of the 442 subjects thought that medical work was very risky. There were 381(86.2%) health workers who think they have more work pressure than before. There were 361(81.7%) health workers who thought that they can accept the risk of exposure to or care for patients with 2019-nCoV. Only 52 (11.8%) health workers felt the urge to resign. Other details are shown in Table 2.

**Table 2.** Impact of 2019-nCoV outbreak on health workers in China (*n* = 442)

Item	*n*	Percentage
1. My work puts me at great risk.	395	89.4
2. I feel more pressure at work than before.	381	86.2
3. I can accept the risk of exposure to or care for patients with the 2019-nCoV.	361	81.7
4. I am afraid of being infected with the 2019-nCoV.	334	75.6
5. I can ensure that my protective measures are effective enough.	207	46.8
6. If I am infected with the 2019-nCoV, I am confident that I can recover.	336	76.0
7. The 2019-nCoV outbreak makes me feel the urge to resign.	52	11.8
8. Because of working in the hospital, my family or friends are worried that they might get infected through me.	280	63.3
9. People stay away from me because of the nature of my work.	144	32.6

### Results of high arousal score and its influencing factors

The average score of high arousal dimension was 5.15 (s.d. = 4.71), and the median score was 4.0 (IQR 2.0, 7.0). Univariate analysis showed that there were significant differences in high arousal scores among different age groups of health workers. Health workers aged 46–55 showed the highest score, while health workers aged less than 25 showed the lowest score. There were critical statistical differences in high arousal scores of genders (male 3.0 *vs*. female 5.0, *P* = 0.077) and whether being quarantined (being quarantined 6.0 *vs*. not being quarantined 4.0, *P* = 0.068) in different groups. The high arousal score of health workers in males was higher than that in females. The quarantined health workers have a higher score for high arousal (Table 1). Multiple regression analysis showed that there were critical statistical differences in high arousal scores among different age groups (male 3.0 *vs*. female 5.0, *P* = 0.075) (Table 3).

**Table 3.** Multiple linear regression with high arousal score as dependent variable

Variables and intercept	*β*	Std error	*t* value	*P* value
Intercept	5.365	2.174	2.467	0.014
Age	0.106	0.304	0.349	0.727
Quarantined	−1.431	0.904	−1.583	0.114
Gender	1.229	0.689	1.785	0.075

### Results of IES score and its influencing factors

The average score of IES was 15.26 (s.d. = 11.23), and the median score was 13.5 (IQR 7.0, 21.0). The results of univariate analysis showed that there were significant differences in IES scores among different age groups and whether being quarantined groups. The older the health workers, the higher the IES score. The IES score of health workers ⩾56 years old was the highest, which was 29.0 (IQR 16.5, 32.5). The IES score of health workers ⩽25 years old was the lowest, which was 11.0 (7.75, 26.0). The quarantined health workers have a higher IES score (Table 1). The univariate analysis showed that in several dimensions of IES, the avoidance score of the quarantined health workers and the total score of IES had significant statistical differences. The score of high arousal dimension has critical statistical significance, while the score of intrusion dimension has no statistical difference (Table 4). The results of multiple regression analysis showed that only the IES scores of whether being quarantined (being quarantined 16.0 *vs*. not being quarantined 13.0, *P* = 0.021) had significant statistical differences (Table 5).

**Table 4.** Univariate analysis of the scores of each dimension of IES and whether being quarantined

Items	Avoidance	Intrude	High arousal	IES
Quarantined	9.0 (4.0,14.0)	10.0 (5.0,13.0)	6.0 (3.0,9.0)	16.0 (11.0,27.0)
Not being quarantined	6.0 (3.0,9.0)	8.0 (6.0,10.0)	4.0 (1.0,7.0)	13.0 (7.0,20.0)
*Z*	−2.674	−1.282	−1.826	−2.033
*P*	0.008	0.200	0.068	0.042

**Table 5.** Multiple linear regression with IES score as the dependent variable

Variables and intercept	*β*	Std error	*t* value	*P* value
Intercept	24.717	4.484	5.513	<0.001
Age	0.060	0.718	0.084	0.933
Quarantined	−4.958	2.149	−2.307	0.021

## Discussion

In this study, most health workers felt that they and their families were at increased risk of infection and increased stress at work. Some health workers feel a certain degree of pressure on the epidemic and 8% of health workers have the idea of quitting. However, health workers are trying to overcome this difficulty, since the 2019-nCoV outbreak, countless health workers have voluntarily signed up to the front line of the fight against the epidemic to treat infected patients at the risk of being infected themselves. As of 8 March 2020, 42 600 health workers in China have gone to Hubei and Wuhan to provide medical assistance, including 19 000 intensive care health workers [‘http://www.nhc.gov.cn/xcs/s3574/202003/a54a40ae28764f3581f36cc31204433c.shtml’, 08 March 2020]. This reflects the great cohesion and unity of the Chinese medical team. A total of 76.0% of health workers believe that they can recover effectively if they are infected. This shows that medical staff have a more correct and objective understanding of 2019-nCoV, and are not afraid of the disease. Secondly, our study found that only 46.8% of health workers thought they were protected effectively. It is reported that more than 2000 medical workers have been infected while caring for 2019-nCoV infected patients [‘http://www.nhc.gov.cn/wjw/mtbd/202003/3a5385c25255461cae9467060bfef27b.shtml’, 12 March 2020]. This reflects that the healthcare facilities still need to strengthen the supply of medical protection resources, ensure an adequate supply of masks, goggles and protective clothing, and increase protection training for medical workers at the same time.

The IES score represents the severity of the subjects' response to stressful events. The higher the IES score, the stronger the individual response to the event [12, 13]. In this study, the average score of IES was 15.26 (s.d. = 11.23), and the median was 13.5 (IQR 7.0, 21.0), which was at a mild level. This score is less than the 34.8 (s.d. = 19.7) score of Chong *et al*. [7] on the IES survey of Chinese medical workers during the SARS outbreak in 2003. The result was also significantly lower than Lee's score of 24.32 for medical workers during the MERS epidemic [9]. However, the IES score increased with age. Health workers ⩾56 years old had the highest IES score of 29.0 (IQR 16.5, 32.5), which was in the middle level. This may be related to the post-traumatic stress disorder caused by health workers ⩾56 years old when they experienced SARS in 2003 [14–18]. This suggests that the hospital can provide psychological counselling and support to health workers whose age is ⩾56 years old. For the health workers who have been quarantined are in the clinical front line of direct contact with infected patients, although their IES scores are relatively high, they are still at a mild level. Policy makers should pay more attention to the quarantined medical workers [19]. The high arousal score reflects the degree of emotional response and anxiety level of the subjects corresponding to stressful events. In this study, the average score of high arousal dimension was 5.15 (s.d. = 4.71), and the median score was 4.0 (IQR 2.0, 7.0). The results of this study show that the overall impact of the 2019-nCoV outbreak on health workers is at a mild level. The review of the literature above shows that the IES scores of health workers in this study are lower than those in SARS and MERS outbreaks. This reflects that Chinese health workers have good psychological coping ability in the face of public health emergencies. The reasons may include two points. First, it has been 17 years since the outbreak of SARS in 2003. In these 17 years, China's medical level and ability to deal with public health emergencies have been greatly improved. Secondly, 2019-nCoV and SARS both belong to coronavirus, and the outbreak time, source of infection, infectivity and CT images are similar to SARS to a certain extent [20–23]. Only 6% of the subjects in this study experienced the SARS epidemic in 2003. However, during the 2019-nCoV outbreak, a considerable number of medical team leaders and decision-makers experienced the SARS epidemic in 2003, which has reliable reference value for scientific, effective and rapid control of the 2019-nCoV epidemic. However, the above explanations are only the authors' personal point of view, and the specific reasons still need more in-depth study in the future.

Through regression analysis, our study observed that compared with the unquarantined health workers, the quarantined health workers had higher scores of avoidance dimension, high arousal and IES. First, quarantined medical workers are required to be quarantined for 14 days. During the quarantine period, they are unable to go out or communicate face-to-face with people. They can only be alone and emotionally lonely. Next, quarantined health workers will continue to get information about the epidemic from the Internet, television and other channels, but they are unable to help patients directly. In the face of the high level of psychological stress of the quarantined health workers, managers should pay attention to this part of the population and carry out online psychological counselling and counselling. However, for the health workers who are in contact with diagnosed or suspected patients with 2019-nCoV, their high arousal score and total IES score have not increased significantly, which may be related to the fact that this part of the population devotes all their energies to the treatment of patients and obtains a certain degree of professional satisfaction at work.

In this study, psychological measurements were carried out on medical workers during the outbreak of 2019-nCoV. Therefore, it can reflect the immediate psychological state of medical workers more truly and objectively. Secondly, this study discusses the influencing factors of the psychological state of medical workers, which can provide targeted psychological intervention to specific groups of people. However, this study also has some limitations. Firstly, due to the busy work of the medical workers who fight directly in the clinical front line, there are fewer subjects who can be directly contacted with diagnosed patients in this study. Secondly, this study uses electronic questionnaire to collect data, all the contents are self-report of the subjects and this is a convenient sample, there may be a certain degree of deviation. Thirdly, the psychological state of health workers in this study is only the current temporary state. If the subjects had a certain degree of anxiety, depression or job burnout, it may have a certain impact on the results of this study. Finally, this study is a cross-sectional study, the long-term mental state has not been followed up.
